# Semi-Automated Training of Rat Ultrasonic Vocalizations

**DOI:** 10.3389/fnbeh.2022.826550

**Published:** 2022-03-04

**Authors:** Aaron M. Johnson, Charles Lenell, Elizabeth Severa, Denis Michael Rudisch, Robert A. Morrison, Adrianna C. Shembel

**Affiliations:** ^1^Department of Otolaryngology-Head and Neck Surgery, New York University Grossman School of Medicine, New York, NY, United States; ^2^Department of Surgery, University of Wisconsin-Madison, Madison, WI, United States; ^3^Department of Communication Sciences and Disorders, University of Wisconsin-Madison, Madison, WI, United States; ^4^Department of Speech Language and Hearing, School of Behavioral and Brain Sciences, UT Dallas, Dallas, TX, United States; ^5^Department of Otolaryngology-Head and Neck, Voice Center, UT Southwestern, Dallas, TX, United States

**Keywords:** ultrasonic vocalizations, voice, automated training, operant conditioning, rat

## Abstract

Rats produce ultrasonic vocalizations (USVs) for conspecific communication. These USVs are valuable biomarkers for studying behavioral and mechanistic changes in a variety of diseases and disorders. Previous work has demonstrated operant conditioning can progressively increase the number of USVs produced by rats over multiple weeks. This operant conditioning paradigm is a useful model for investigating the effects of increased laryngeal muscle use on USV acoustic characteristics and underlying central and peripheral laryngeal sensorimotor mechanisms. Previous USV operant conditioning studies relied on manual training to elicit USV productions, which is both time and labor intensive and can introduce human variability. This manuscript introduces a semi-automated method for training rats to increase their rate of USV production by pairing commercially available operant conditioning equipment with an ultrasonic detection system. USV training requires three basic components: elicitation cue, detection of the behavior, and a reward to reinforce the desired behavior. With the semi-automated training paradigm, indirect exposure to the opposite sex or an olfactory cue can be used to elicit USV production. The elicited USV is then automatically detected by the ultrasonic acoustic system, which consequently triggers the release of a sucrose pellet reward. Our results demonstrate this semi-automated procedure produces a similar increase in USV production as the manual training method. Through automation of USV detection and reward administration, staffing requirements, human error, and subject behavioral variability may be minimized while scalability and reproducibility are increased. This automation may also result in greater experimental flexibility, allowing USV training paradigms to become more customizable for a wider array of applications. This semi-automated USV behavioral training paradigm improves upon manual training techniques by increasing the ease, speed, and quality of data collection.

## Introduction

Rats produce ultrasonic vocalizations (USVs) for conspecific communication of affective states (Brudzynski, 2009). USVs are produced within the larynx and require fine motor control of the intrinsic laryngeal muscles (Kelm-Nelson et al., 2018). These USVs are valuable biomarkers for studying behavioral and mechanistic changes in a variety of diseases and disorders, such as Parkinson’s disease, autism, and age-related voice disorders (Johnson et al., 2015; Caruso et al., 2020; Krasko et al., 2021).

Previous work has demonstrated operant conditioning can progressively increase the number of USVs produced by rats over multiple weeks (Johnson et al., 2011). This operant conditioning paradigm is a useful model for investigating the effects of increased laryngeal muscle use on USV acoustic characteristics and underlying central and peripheral laryngeal sensorimotor mechanisms (Johnson et al., 2013; Lenell et al., 2019; Krasko et al., 2021; Shembel et al., 2021). However, previous work relied on manual training by hand to elicit USV productions (Johnson et al., 2011). While this method is effective in training USVs, it is time and labor intensive and can introduce human variability. Rewarding by hand can only be done one rat at a time and requires at least one human tester to monitor USVs. One solution to these shortcomings is to automate USV training, which would allow for simultaneous monitoring of multiple animals while keeping temporal and stimuli-response criteria consistent. The goals of this study were to develop an automated operant conditioning procedure for training rats to increase their rate of USVs and to determine whether outcomes of the automated training paradigm were equivalent to manual hand training.

## Materials and Equipment

**FIGURE 1:**
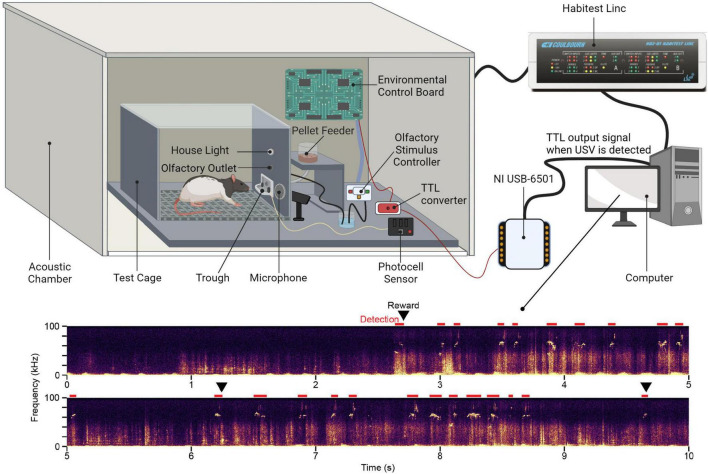
Schematic of the equipment used for semi-automated ultrasonic vocalization training Figure created with BioRender.com.

The semi-automated system consists of operant conditioning hardware and software for reinforcing USV production and an ultrasonic acoustic monitoring system for detecting USVs. Table 1 contains a detailed list of all equipment and Figure 1 presents a schematic of the primary equipment.

**TABLE 1:** Detailed list of equipment and materials.

*Name*	*Product number*	
**Operant conditioning system (Coulbourn instruments)**		
HABITEST Linc	H02-08	
Environment control board	H03-04	
Modular test cage - rat	H10-11R-TC	
Non-shock floor for rat test cage	H10-11R-TC-NSF	
Assorted wall panel set – rat	H90-00R-M-KT01	
House light rat (Led)	H11-01-R-LED	
Pellet trough led - rat	H14-01R-LED	
Single photocell sensor	H20-94	
Pellet feeder, 45 mg - Rat	H14-23R	
Olfactory stimulus controller	H15-03	
Olfactory evaporation chamber	H15-20	
Fan module- rat	H29-05R	
Tygon 2375 1/8” Id -1/16” Wt	724120	
5 Volt TTL - 24 Volt converter 110v	H91-24	
Spare to switch input adapter	H91-16-SPARE-IN	
Olfactory stim inj panel - rat	H15-01R	
H10-24 with acoustic liner	H10-24A	
Graphic state notation 4 software	GS4.0	

***Name***	***Product number***	

**Ultrasonic recording and detection system (Avisoft bioacoustics)**		
Avisoft RECORDER	10301	
UltraSoundGate 816H	34171	
Condenser ultrasound microphone CM16/CMPA	40011	
Calibrated 40 kHz reference signal generator	60105	

***Name***	***Product number***	

**Air supply for automated scent delivery (Airgas)**		
Regulator analytical first stage 0-50 PSI delivery CGA346 3500 PSI Inlet 1/4” Male NPT	Y11244B346-AG	
Flowmeter standard 65 mm 20 HF Flow 60 PSI teflon frame stainless steel float 1/8” female NPT	Y21T654-AG	
Air breathing Gr D Size 125 CGA 346	AI B125	
Adapters	Y99593HCB4F-AL, Y99480094-AG, Y994801220-AG, Y9926115-AG	

***Name***	***Manufacturer***	***Product Number***

**Additional equipment**		
Dustless precision pellets, 45 mg, rodent purified diet	Bio-Serv	F0021
24-Channel, 8.5 mA, digital I/O device	National instruments	NI USB-6501
BNC cables and adapters to transmit TTL signal from NI USB-6501 to Coulbourn H91-24	Digi-Key	assorted

### Operant Conditioning Hardware and Software

The automated operant conditioning system consists of commercially available hardware and software from the HABITEST Modular Behavioral Test System (Coulbourn Instruments, Holliston, MA, United States). A single setup is comprised of a modular test cage housed within a soundproof acoustic chamber to allow for simultaneous training of multiple animals in the same room. The modular test cage contains a pellet feeder and trough to deliver a nutritional pellet reward (F0021, Bio-Serv, Flemington, NJ, United States). Pellet reward retrieval is monitored using a photocell sensor on the trough. To elicit USVs, rats are either indirectly exposed to a conspecific of the opposite sex or an olfactory stimulus controller delivers an air puff with the scent of an animal of the opposite sex via a tube connected to an outlet located immediately above the trough. All equipment is connected to and controlled by an environmental control board that, in turn, is connected to a Habitest Linc. The Linc is connected to a computer and allows the user to design and carry out experiment protocols through the Graphic State and Graphic StateRT software programs, respectively.

### Ultrasonic Acoustic Monitoring

Test cages are individually monitored using an ultrasonic acoustic system consisting of a condenser ultrasound microphone (CM16/CMPA, Avisoft Bioacoustics, Glienicke, Germany) connected to a USB recording interface (UltraSoundGate 816H, Avisoft Bioacoustics) which powers the microphone and performs the analog-digital conversion. Each cage is independently monitored for USV events using RECORDER software (Avisoft Bioacoustics).

When a USV is detected by the Avisoft program, a TTL signal is sent through a USB digital I/O interface (NI USB-6501, National Instruments, Austin, TX, United States) and routed to the environmental control board within the testing apparatus via a 5V to 24V converter, thereby providing a trigger to dispense the pellet reward. Additionally, the Avisoft system records acoustic files from each training session for later offline acoustic analysis. Commonly used acoustic analysis programs in our laboratory include Avisoft SASLab Pro and DeepSqueak, a MATLAB-based software for USV detection and acoustic analysis (Coffey et al., 2019).

### Reward Pellet

Because the pellet reward plays a large role in reinforcing vocalization behavior in our paradigm, it is essential that the appeal of the pellet does not diminish over multiple weeks of training. Sucrose-only pellets were initially used as a reward, as these types of pellets have previously demonstrated effectiveness in eliciting target behaviors (Leenaars et al., 2019). Nutritional sucrose pellets (Bio-Serv Dustless Precision Pellets, 45 mg unflavored) were also considered. Nutritional pellet rewards provide a more complex and nutritionally dense option that may be more appealing to feed-restricted animals and reduces reliance on the need for grain pellet diets between training sessions. Nutritional sucrose pellets were ultimately chosen over the sucrose-only pellets as animals showed preference for the latter. A mix of sucrose and nutritional pellets (e.g., 1:3) may also be used to further motivate rats to seek the reward.

### Animals

All experiments were approved by the Institutional Animal Care and Use Committee at the New York University Grossman School of Medicine. Our manual USV training paradigms have been successful in Long Evans (Charles River, Wilmington, MA, United States) and Fischer 344 x Brown Norway rats (National Institute on Aging rodent colony). Therefore, we used Long Evans rats (12 weeks old; male) to test the automated training. All rats were kept on a 12/12 reverse light cycle and trained during their dark cycle when rats are most active. Water was provided *ad libitum*. Access to food was restricted, with grain pellet feed removed from the home cage prior to the days training was conducted to motivate interest in the nutritional sucrose pellet reward. Rats in the experimental group were restricted to nutritional sucrose pellet rewards received during training. Rats in the control group received a small quantity of nutritional sucrose pellets in their home cage in addition to grain feed pellets at the end of each training day. On non-training days, rats were given unrestricted access to grain feed. Leftover grain feed was removed in the morning before the next training session was conducted. Rats were weighed three times a week and were supplemented with grain feed if their weight dropped below 80% of their baseline.

## Materials and Methods

### Protocol Overview

**FIGURE 2:**
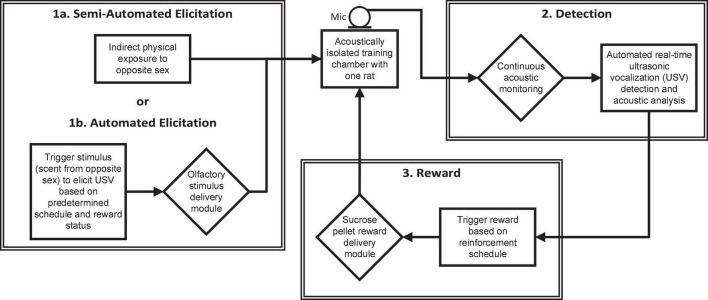
Diagram of the semi-automated ultrasonic vocalization (USV) training procedure indicating the equipment (diamonds) and processes (rectangles) involved in the elicitation, detection, and reward of USVs.

**FIGURE 3:**
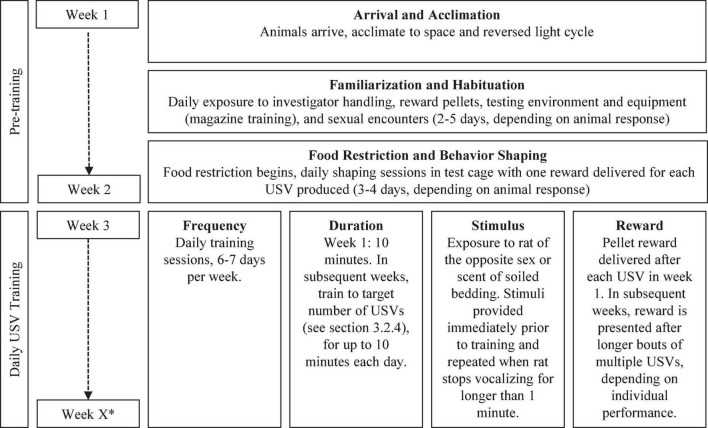
Overview of pre-training and behavioral training timeline. *Total number of weeks of training is determined by the experimental goal.

Ultrasonic vocalizations training requires three basic components: elicitation cue, detection of the behavior, and reward (Figure 2). In the manual hand training, the elicitation cue was direct physical exposure to the opposite sex. This cue was quite salient – perhaps overwhelmingly so, as it often distracted the animal from the reward. With the automated training paradigm, indirect exposure to the opposite sex (with the animals separated by a wire mesh) or an olfactory cue can be used to elicit initial vocalizations. When a USV is produced in response to the elicitation cue, it is detected by the ultrasonic acoustic system and a sucrose pellet is delivered through the trough in the HABITEST Modular Behavioral Test System cage. An overview of the training procedures is shown in Figure 3.

### Pre-training Considerations

#### Acoustic Environment

One general consideration when preparing the physical space for USV training is to check for extemporaneous ultrasonic noises that could inadvertently trigger USV detection. Some possible sources of ultrasonic noise include computer equipment (e.g., monitors), cell phones, motion detectors, and HVAC vents. Rodent claws on metal gratings, siding, and cage clamps can also trigger USV detections. If multiple rats are present in the training room, individual training apparatuses need to be acoustically isolated to ensure each animal is only rewarded for their individual USV productions. The acoustic isolation chamber described in this experiment provides sufficient acoustic isolation, as would acoustic foam positioned between cages.

#### Familiarization and Habituation

To control for novelty effects which could influence USV elicitation and training (Kelm-Nelson et al., 2016), several pre-training familiarization and habituation procedures are used.

##### Handling

Familiarization with training staff can be achieved by handling the animals daily and by transporting them from their home cage to the test cage.

##### Reward Pellets

To familiarize the animals with the reward pellets, a small dish with the pellets can be placed in the animals’ home cage a few days before training begins.

##### Testing Environment

Placing animals in the test cage several times before formal USV training begins to familiarize animals with the new environment can increase initial USV rate.

##### Sexual Encounters

Introducing male rats to females in estrus increases the chance of the males expressing interest in the opposite sex pairing, which can also improve olfactory USV elicitations. In our previous studies and pilot testing, we found that young male rats (under the age of 12 months) typically mount with one or two brief interactions with a receptive female, whereas older male rats (over the age of 12 months) may require several consecutive days of exposure to estrus females before an attempt at copulation occurs. Additionally, we have found up to 20-50% of male rats do not express interest in female rats and do not vocalize with the female rat exposure. These rats are excluded from training procedures.

##### Magazine Training

Magazine training can be utilized to familiarize experimental animals with a novel environment (test cage) prior to the start of formal USV training. In magazine training, the rat is placed in the operant conditioning chamber and presented a reward at random intervals (average 1-3 pellet per minute). Magazine training typically takes place for 10-30 min per day for 2-5 days, depending on the response from each rat.

##### Transport

If animals need to be transported from their colony to the training site, allow the animals to remain in their home cage for 10-15 min to habituate to the environment prior to each training session.

#### Behavior Shaping

The goal of behavior shaping is to reward approximate target behaviors. During shaping, each rat is rewarded once for every bout of USVs. Because USV production is influenced by strain, age, and sex of the rat, each bout may consist of a single USV or a string of several USVs in rapid succession.

### Training Schedule

#### Session Timing

Training sessions always occur during the active (dark portion) of the light cycle. In our experience, the specific time of day the training session is conducted within the active portion of the cycle may influence USV production. To minimize any potential influence of time of day, we conducted training during the first 4 h of the dark portion of the cycle.

#### Session Duration

**FIGURE 4:**
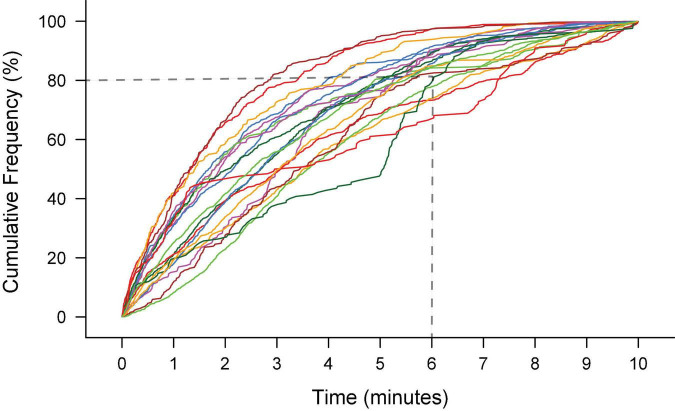
The ultrasonic vocalization (USV) productions during 10 min of recording of 20 female Long-Evans rats elicited by an introduction to a male rat. Each line is the cumulative frequency of USVs for an individual rat. The majority (16/20) rats produced 80% of their total number of USVs within the first 6 min of recording. The total number of USVs produced ranged from 100 – 700.

In our previous manual studies, we have demonstrated rodents vocalize throughout sessions with a duration of 10 min but do not perform consistently in longer sessions. For example, following initial introduction to male rats, most female rats produce 80% of their vocalizations within the first 6 min of a 10-min recording session (Figure 4; Lenell and Johnson, 2021). If longer duration of training is preferred with the semi-automated training, water *ad libitum* is recommended, as rats will get thirsty and lose motivation.

#### Number of Sessions per Week

Since weekend breaks from USV training can adversely affect consistency and rate of training responses, 6-day a week training schedules are recommended.

#### Daily Training Target

Because of variability in baseline USV production rates amongst rats, it is advisable to individualize weekly USV training targets based on each individual rat’s baseline performance. For example, in a prior USV manual training study using Fisher 344 × Brown Norway male rats, the young (9-month-old) cohort produced a range of 33-150 USVs per session during their first week of 10-min USV training. In this example, a predetermined goal of 100 USVs per training session would be unattainable for one rat to produce while being an easy target for another rat.

Our training protocol has focused on the progression of USV production rate over a fixed amount of training duration. In manual training, production of USVs rapidly increased during the first four weeks of USV training and then steadily increased thereafter. USV training targets with automated training can be increased by 50% each week for the first 4 weeks and then by 20% in additional weeks of training. If rats are not able to achieve their individual goal for the week, the goal remains unchanged for the following week until the target percentage is achieved across at least 2 consecutive sessions.

### Elicitation

USVs can be elicited using either manual presentation of a rat of the opposite sex or using soiled bedding as an olfactory stimulus. In both methods, prior to testing, animals should be familiarized with the scent and appearance of opposite sex rats to combat sexual naivety and increase valuation of the stimulus animals or olfactory stimulus. Once vocal training begins, the elicitation occurs within the first couple of weeks and is then phased out once the animals make the association between vocalizations and the sucrose reward. In manual USV training, USVs can be elicited by briefly exposing sexually experienced rats to the opposite sex. While this method robustly elicits USVs, it requires manual presentation and removal of stimulus animals. To bypass this, other labs have found olfactory exposure to the opposite sex can elicit USV production in sexually experienced male rats (Ciucci et al., 2008). Preliminary results from our lab also confirms that olfactory cues can elicit USVs, although the response is less robust than physical presentation of a stimulus animal.

For the olfactory stimulus, one week’s worth of soiled bedding can be removed from female home cages and soaked in water. The water can then be used as an olfactory cue by placing the liquid through an olfactory module system (Coulbourn Instruments, Harvard Bioscience, Boston, MA, United States). The module propels air through the system using a compressed air tank with a low pressure (1-5 PSI) and low flow rate of (5 SCFH or less). This configuration delivers a one-second puff of air with the olfactory stimulus into the test chamber during each training session. To maintain novelty of the olfactory stimuli, soiled bedding should be presented from different animals. To prevent competing olfactory stimuli, bedding should be removed from the tray underneath the testing chamber after each session. One caveat of this procedure is that the brief olfactory cue may elicit USVs for some rats, however, low vocalizing rats may need physical presentation of the stimulus animal to elicit USVs.

### Ultrasonic Vocalizations Detection Parameters

**FIGURE 5:**
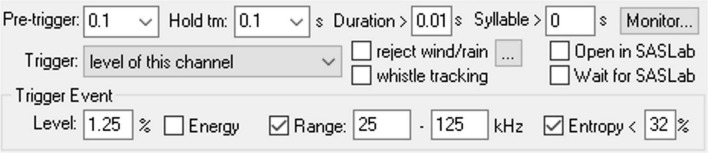
Screenshot of the configuration settings in Avisoft RECORDER for automatic detection of ultrasonic vocalizations.

The Avisoft RECORDER software configuration allows for automatic USV detection based on several different USV acoustic parameters: signal amplitude, duration, frequency range, and amount of entropy. Additionally, a USV bout can be counted as a single event by adjusting the hold time parameter, which holds the current trigger as active for a defined duration between each detected USVs. The specific values for the detection parameters used in our preliminary studies are shown in Figure 5. These values may need to be adjusted to account for USV production variability across animals and extemporaneous environmental noise. The configuration can be tweaked and tested using the USV real-time monitoring tool and running the detection on pre-recorded sound files with the “run from.wav file” option. With multi-cage setups, each microphone should be calibrated to the same intensity level using an ultrasonic signal generator (Avisoft Bioacoustics) to maintain intensity consistency between recording hardware.

### Reward

For initial learning effects, each vocalization should be paired with one sucrose pellet. After the animal is consistently making the association between USV production and reward, sucrose reward presentations can be presented after several bouts of vocalizations (variable reward).

#### Visual Cue for Reward

The preinstalled house light in the operant testing chamber can be illuminated as a means of providing a visual cue associated with the pellet reward and the start of the vocalization training session; however, we found that vocalizations occurred immediately upon placement of the animal into the testing chamber regardless of illumination status. Therefore, the house light can remain illuminated throughout the training to facilitate visual tracking of the animal’s movement without concern it will influence USV production.

## Results

**FIGURE 6:**
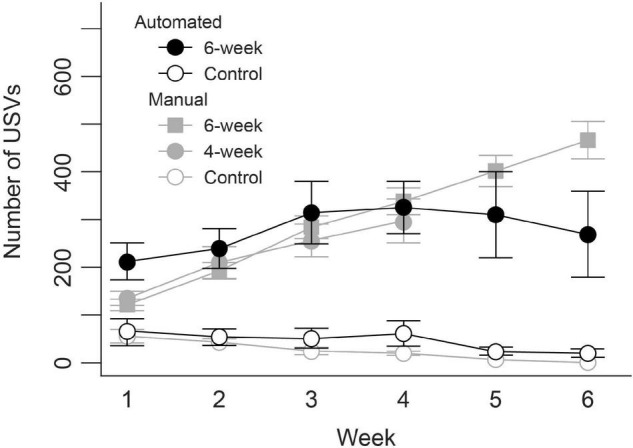
Representative results of the weekly increase in ultrasonic vocalization production during semi-automated training compared to previous manual training.

Figure 6 compares results from our previous work using manual USV training to animals trained using the semi-automated procedure. In the manual training, USV production steadily increased across 4 and 8 weeks of manual training in 16 male Long Evans rats. The semi-automated procedure resulted in a similar increase in the number of USVs produced over the first 4 weeks of training. However, increases in performance in rats trained using the semi-automated procedure began to flatten during the fifth week and then decreased in week 6. The untrained control groups in both the manual and semi-automated training paradigms produced a minimal number of USVs throughout the training periods.

## Discussion

The move to automate the USV training process brings several key advantages over commonly used manual training techniques. Automated training reduces staffing requirements while increasing the number of animals that can be trained simultaneously. Automated training also allows for multiple and longer training sessions throughout a day, providing an opportunity to increase the total number of vocalizations elicited compared to manual training. Furthermore, automating the reward process provides the animal with a more consistent behavioral experience and less human exposure, reducing inherent human variability with manual presentation of rewards. Together, these advantages position automated USV training to provide a higher level of scalability and consistency than traditional manual training, allowing for increased experimental bandwidth and ultimately faster, more reproducible data collection.

Automated training also allows for greater experimental flexibility. Here, we outline the use of olfactory and visual cues to enhance behavioral training. Other experimental designs could conceivably benefit from precisely timed administration of cues in different sensory modalities. For example, reward conditions during training could be tied to specific sound cues, or operant conditioning paradigms could automatically apply opposing cues to reinforce target behaviors and dissuade others. Moreover, the automatic detection of USVs employed here could allow for training of specific vocalization characteristics. By systematically changing USV detection criteria over the course of behavioral shaping, animals could be trained to perform specific types of vocalizations. For example, during the behavioral shaping period detection criteria for duration of USVs could be steadily lengthened as performance increases, preferentially rewarding and reinforcing longer and longer vocalizations until a target duration is met. Similar shaping paradigms could potentially reinforce vocalizations of a specific frequency, intensity, or type (e.g., frequency modulated versus flat tone) by slowly narrowing USV detection criteria for frequency, amplitude, bandwidth, and sinuosity. Our preliminary results with this new semi-automated training yielded similar progressive increases in the number of overall USVs produced as previously found in manual training. However, it is important to note that while performance continued to increase over 8 weeks in the manual training, there was a dip in performance after 6 weeks with the semi-automated training. These differences need to be considered when developing and conducting experimental designs and may indicate an inherent limitation of long-term automated training, or that a change in behavioral training paradigm around week 6 is needed to counteract this downturn in performance.

Through automation of USV detection and reward administration, staffing requirements, human error, and subject behavioral variability may be minimized while scalability and reproducibility is increased. This automation may also result in greater experimental flexibility, allowing USV training paradigms to become more customizable for a wider array of applications. Here, we outline a semi-automated USV behavioral training paradigm that improves upon manual training techniques by increasing the ease, speed, and quality of data collection.

## Data Availability Statement

The raw data supporting the conclusions of this article will be made available by the authors, without undue reservation.

## Ethics Statement

The animal study was reviewed and approved by the Institutional Animal Care and Use Committee at the New York University Grossman School of Medicine.

## Author Contributions

AJ, AS, and CL contributed to conception and design of the study. AS, CL, DR, ES, and RM conducted the training trials. All authors wrote the sections of the manuscript and contributed to the manuscript revision, read, and approved the submitted version.

## Conflict of Interest

The authors declare that the research was conducted in the absence of any commercial or financial relationships that could be construed as a potential conflict of interest.

## Publisher’s Note

All claims expressed in this article are solely those of the authors and do not necessarily represent those of their affiliated organizations, or those of the publisher, the editors and the reviewers. Any product that may be evaluated in this article, or claim that may be made by its manufacturer, is not guaranteed or endorsed by the publisher.
